# Different transcriptional regulatory activities of Mycobacterium bovis and Mycobacterium tuberculosis PhoPR systems

**DOI:** 10.1099/acmi.0.001087.v3

**Published:** 2026-01-28

**Authors:** Jose Maria Urtasun-Elizari, Ruoyao Ma, Hayleah Pickford, Damien Farrell, Viktor Perets, Jesus Urtasun-Elizari, Gabriel Gonzalez, Chie Nakajima, Yasuhiko Suzuki, Apoorva Bhatt, David E. MacHugh, Stephen V. Gordon

**Affiliations:** 1 UCD School of Veterinary Medicine, University College Dublin, Belfield, Dublin, D04 V1W8, Ireland; 2 School of Biosciences and Institute of Microbiology and Infection, University of Birmingham, Birmingham B15 2TT, UK; 3 MRC LMS, Institute of Clinical Sciences, Faculty of Medicine, Imperial College London, London, UK; 4 Institute for Vaccine Research and Development, Hokkaido University, Kita 21, Nishi 11, Kita-ku, Sapporo, 001-0021, Japan; 5 International Institute for Zoonosis Control, Hokkaido University, Kita 20, Nishi 10, Kita-ku, Sapporo, 001-0020, Japan; 6 UCD School of Agriculture and Food Science, University College Dublin, Belfield, Dublin, D04 V1W8, Ireland; 7 UCD Conway Institute of Biomolecular and Biomedical Science, University College Dublin, Belfield, Dublin, D04 V1W8, Ireland; 8 UCD One Health Centre, University College Dublin, Belfield, Dublin, Ireland

**Keywords:** bovine tuberculosis, host adaptation, *Mycobacterium tuberculosis* complex (MTBC), PhoPR, RNA sequencing, tuberculosis

## Abstract

Tuberculosis (TB) is an infectious disease that affects humans and animals. The pathogens that cause TB belong to the *Mycobacterium tuberculosis* complex (MTBC), with *M. tuberculosis* and *Mycobacterium bovis* as the main representatives of human- and animal-adapted strains, respectively. One key genetic regulator of the MTBC members is the PhoPR system, which controls many processes, including the stress response, lipid metabolism and pathogenesis, among others. Previous studies identified a key G71I substitution in the *M. bovis* PhoR orthologue relative to *M. tuberculosis* PhoR and suggested that PhoPR might be non-functional in animal-adapted strains, but recent work has highlighted the functionality of PhoPR in * M. bovis* despite the G71I substitution. Here, we compare the transcriptional effects of the PhoPR system of *M. tuberculosis* H37Rv and *M. bovis* AF2122/97 on an *M. bovis* AF2122/97 Δ*phoPR* knockout background. Our results show common patterns of gene expression between the two orthologues, but also clear differences in the expression of rubredoxin genes and lipid biosynthetic loci. This work adds to the evidence that the PhoPR system is indeed functional in *M. bovis* and suggests that PhoPR controls differential transcriptional programmes that are important in the adaptation to human or animal hosts.

## Data Summary

The authors confirm that all supporting data, software and protocols used have been provided within the article or through supplementary data files. The RNA-sequencing data are available at the NCBI Sequence Read Archive under the BioProject ID PRJNA1129457. The raw sequencing files have been deposited under the following accession numbers: SRR29637966, SRR29637965 and SRR29637962 (*M. bovis* wild-type replicates); SRR29637961, SRR29637960 and SRR29637959 (*M. bovis* Δ*phoPR* knockout replicates); SRR29637958, SRR29637957 and SRR29637956 (*M. bovis* Δ*phoPR*::phoBovis complement replicates); and SRR29637955, SRR29637964 and SRR29637963 (*M. bovis* Δ*phoPR*::*phoTB* complement replicates). The custom scripts used to run the analysis are available on the public GitHub repository at https://github.com/jurtasunel/PhoPR_manuscript.

## Introduction

Tuberculosis (TB) is a disease of humans and animals caused by genetically similar bacterial pathogens that constitute the *Mycobacterium tuberculosis* complex (MTBC) [1,2]. Depending on their host tropism, the MTBC can be classified into two main groups, the human-adapted or the animal-adapted species, with *M. tuberculosis* and *Mycobacterium bovis* as the primary representatives of these host-adapted groups, respectively. Despite high identity at the genomic level, MTBC members exhibit discrete genetic differences that have been postulated to provide the molecular basis for the distinct host-pathogen interactions among different lineages [3,4].

An important locus that shows variation among MTBC lineages encodes the PhoPR system (encoded by *Rv0757* and *Rv0758* in *M. tuberculosis*), which is a two-component signal transduction system (2-CS) that coordinates a variety of processes in MTBC bacilli, including secretion of proteins involved in virulence, lipid biosynthesis, stress responses and intracellular adaptation [5,8]. Moreover, an *M. tuberculosis* knockout in the PhoP response regulator is one of the two attenuating mutations in MTBVAC, the first attenuated TB vaccine candidate to enter clinical trials [9].

A G71I substitution in the PhoR kinase that differentiates *M. bovis* from *M. tuberculosis* was identified by Gonzalo-Asensio *et al*., and it was suggested that the *M. bovis phoR* allele was ‘defective’ compared to the *M. tuberculosis* orthologue [10]. Subsequent analysis of the *M. bovis* PhoPR system by Garcia and colleagues showed that, despite the G71I substitution, the *M. bovis* 04-303 PhoPR system still controlled several transcriptional processes, including response to stress and the regulation of ESAT-6 expression [11,12]. Our previous work supports the findings of Garcia *et al*., where we found extensive differential expression of genes in an *M. bovis* AF2122/97 PhoPR knockout as compared to wild-type, further supporting the hypothesis that the PhoPR system of *M. bovis* is indeed functional [13].

In this work, our aim was to compare the functionality of the *M. bovis* PhoPR system with the *M. tuberculosis* PhoPR orthologue on an *M. bovis* AF2122/97 genetic background. To do this, we complemented an *M. bovis* AF2122/97 *phoPR* knockout with either the PhoPR system of *M. bovis* AF2122/97 or that of *M. tuberculosis* H37Rv, so the two complemented mutants would only differ by the PhoR G71I substitution. We performed transcriptomic analyses to explore differential gene expression between these two systems. Our results show a common regulatory profile between the PhoPR system of *M. bovis* and *M. tuberculosis*, as well as key transcriptional differences in the regulation of genes encoding rubredoxin and lipid metabolic pathways.

## Methods

### Strains and cultures

The parental strain used in this study was *M. bovis* AF2122/97 wild-type. The construction of the *M. bovis* AF2122/97 Δ*phoPR* knockout is described by Urtasun-Elizari *et al*. [13]. The complementation strategy for this knockout is described in the methods section below.

For general culture conditions, mycobacteria were grown in Middlebrook 7H9 liquid broth (Difco) supplemented with 0.05% Tween 80 (Sigma-Aldrich), 0.2% glycerol (Sigma-Aldrich), 0.5% BSA (Sigma-Aldrich), 40 mM sodium pyruvate (Sigma-Aldrich) and 0.085% NaCl. For solid agar medium, 7H11 agar (Difco) was used, supplemented with 0.2% glycerol (Sigma-Aldrich), 0.5% BSA (Sigma-Aldrich), 0.085% NaCl and 40 mM sodium pyruvate (Sigma-Aldrich).

For transformant and mutant selection, the antibiotics used in the media were as follows: hygromycin at a final concentration of 100 µg ml^−1^, kanamycin at 50 µg ml^−1^ and zeocin at 25 µg ml^−1^.

### Complementation of *M. bovis phoPR* knockout mutant

The complementation of the *M. bovis* Δ*phoPR* knockout with either the *M. bovis* or *M. tuberculosis phoPR* orthologue was achieved using a combination of the recombineering method [14] and site-directed mutagenesis.

Briefly, the recombineering method was used to amplify the promoter region and CDS of *M. bovis phoPR*, clone them in separate vectors and merge them by *att* recombination into a final destination vector, in our case, the integrative pDE43-MCZ vector. The destination vector containing the *phoPR* locus of *M. bovis* was then converted into the *M. tuberculosis phoPR* orthologue by mutating codon 71 of *phoR* from ATT to GGT (that codes for the *phoR* G71I variant between *M. tuberculosis* and *M. bovis*) using site-directed mutagenesis. Both plasmids were sent for Sanger sequencing verification and then transformed by electroporation into the *M. bovis* AF2122/97 Δ*phoPR* knockout mutant. The complemented mutants were verified by Sanger sequencing of the *phoPR* locus and qPCR. Table 1 shows the primers used for the cloning of the *phoPR* locus using the Gateway cloning system and the primers used to introduce the *M. tuberculosis* G71I substitution into the *M. bovis phoR* orthologue. The complemented mutants were named *M. bovis* Δ*phoPR*::*phoBovis* and *M. bovis* Δ*phoPR*::*phoTB*.

**Table 1.** Primers used for the *phoPR* cloning

Name	Sequence (5′–3′)
Recombineering	
F1	GGGG**ACAAGTTTGTACAAAAAAGCAGGCT**GTGTCATCGATTCCCAGCAT*
R1	GGGG**ACCACTTTGTACAAGAAAGCTGGGTA**TCACAGTCATTGTGTAATTC*
F2	GGGG**ACAGCTTTCTTGTACAAAGTGGAC**AGGTAACGTTCAACCAATGC*
R2	GGGG**ACAACTTTGTATAATAAAGTTGC**CCTCAGTGATTTCGGCTTTG*
Site-directed mutagenesis	
F	[phos]GACCCCTACCCT**GG**TCATAACCCCG†
R	[phos]CGGCGCCAAGGGCAGCGTGATCTGC

*The sequences in bold represent the *att* recombinogenic sites required for the Gateway cloning. The underlined sequences correspond to the *phoPR* locus.

†The sequence in bold represents the AT to GG mutation from *M. bovis *to *M. tuberculosis*.

### Isolation and purification of mycobacterial RNA

Mycobacterial cultures were grown in 50 ml Middlebrook 7H9 liquid media with rolling until they reached an OD_600_ of 0.6–0.8; for each sample, three independent biological replicates were grown. The cultures were then pelleted by centrifugation at 3,000 ***g*** for 10 min, resuspended in 1 ml QIAzol lysis reagent (Qiagen) and transferred to a 2 ml screw cap tube containing bead-beater glass beads (BioSpec Products). The cells were lysed by bead beating using a MagNA lyser (Roche Diagnostics) for two cycles of 30 s at maximum speed (7,000 r.p.m.). The lysate was then either stored at −80 °C or used immediately for the next extraction step.

1-Bromo-3-chloropropane (Sigma) was added, and the suspension was mixed well and centrifuged at 12,000 ***g*** for 15 min to obtain the aqueous layer containing the nucleic acids. Following this, 1.5 volumes of 100% ethanol were added to the sample, and the RNA extraction and purification were performed with the RNeasy kit (Qiagen) following the manufacturer’s protocol. The RNA concentration and quality were measured using a NanoDrop Spectrophotometer (Thermo Fisher Scientific) and then diluted to a final concentration of 200 ng µl^−1^ in a final volume of 50 µl.

Residual DNA was removed from the RNA samples using the Turbo DNA-free kit (Thermo Fisher Scientific). Five microlitres of buffer and 1 µl of Turbo DNase (2 units of enzyme) were added to each RNA sample and incubated at 37 °C for 30 min, followed by an addition of a further 1 µl of enzyme and another 30 min, with incubation according to the manufacturer’s protocol. The RNA samples were then purified and concentrated using an RNA Clean and Concentrator kit (Zymo Research) following the manufacturer’s instructions.

### Reverse transcription

cDNA was obtained using a High-Capacity cDNA Reverse Transcription Kit (Thermo Fisher Scientific). The purified RNA samples were diluted to a final concentration of 100 ng µl^−1^ in a final volume of 10 µl cDNA, and a reverse transcription master mix was produced with a composition per reaction of 2 µl of buffer, 1 µl of reverse transcriptase, 0.8 µl of 100 mM dNTP mix, 2 µl of random primers and 4.2 µl of MilliQ water (Sigma-Aldrich) according to the manufacturer’s protocol. Ten microlitres of the master mix were added to each 10 µl RNA sample and incubated at 37 °C for 2 h.

### RNA sequencing

RNA sequencing (RNA-seq) was performed by a commercial service provider (Genewiz), including library preparation and Illumina NovaSeq 2×150 bp paired-end sequencing to obtain ~10 million reads per sample. RNA-seq data are available at the NCBI Sequence Read Archive under the BioProject ID PRJNA1129457.

### Bioinformatics analysis

Quality control of the raw sequencing data was performed using the FastQC tool (version 0.11.9) [15] to assess quality and length of the reads, GC content per sequence, overrepresented sequences and presence of adapters, among other parameters. The reads were trimmed using the cutadapt tool (version 3.5) [16] with the following threshold values: number of bases to trim (10 to 15 bp on each side), minimum quality of reads (minimum Phred score of 25) and minimum length of reads (65 bp). The reads were aligned to the *M. bovis* AF2122/97 reference genome (NC_002945.4) using Bowtie2 (version 2.4.4) [17] and processed with SAMtools (version 1.13) [18]. The binary alignment maps of the different samples were merged in a summarizing count matrix using the featureCounts function from the Rsubread package (version 2.18.0) [19]. The count matrix containing all the raw counts can be found on the first sheet of the Supplementary Data file, available in the online Supplementary Material.

The differential expression analysis was performed using the R package DESeq2 (version 1.44.0) [20]. Genes with log_2_ fold change absolute values (|log_2_fc|)>1 and *P*-adjusted values (*Padj*) <0.05 were considered differentially expressed. The *P*-values were adjusted using the Benjamini–Hochberg correction. The volcano plots of each comparison can be found on the third sheet of the Supplementary Data file. The gene categories used for the common transcription profile comparison follow the notation established by Cole *et al*. [21] and were obtained through the Artemis browser (version 18.2.0) from the Wellcome Sanger Institute [22]. Plotting was done using the R packages complexHeatmap (version 2.20.0) [23] and ggplot2 (version 3.5.1) [24]. The gene network plots were done using the open-source software Cytoscape (version 3.10.1) [25]. The Z-scores represent normalized gene expression values; they are calculated by subtracting from each raw count the average expression value of that gene across all samples and then dividing it by the standard deviation. Hence, Z-scores higher or lower than 0 mean that the expression of that gene in that sample is higher or lower, respectively, than the average expression of that gene across all samples.

## Results

### A common transcriptional profile between *M. bovis* Δ*phoPR*::*phoBovis* and *M. bovis* Δ*phoPR*::*phoTB*

**Fig. 1.**
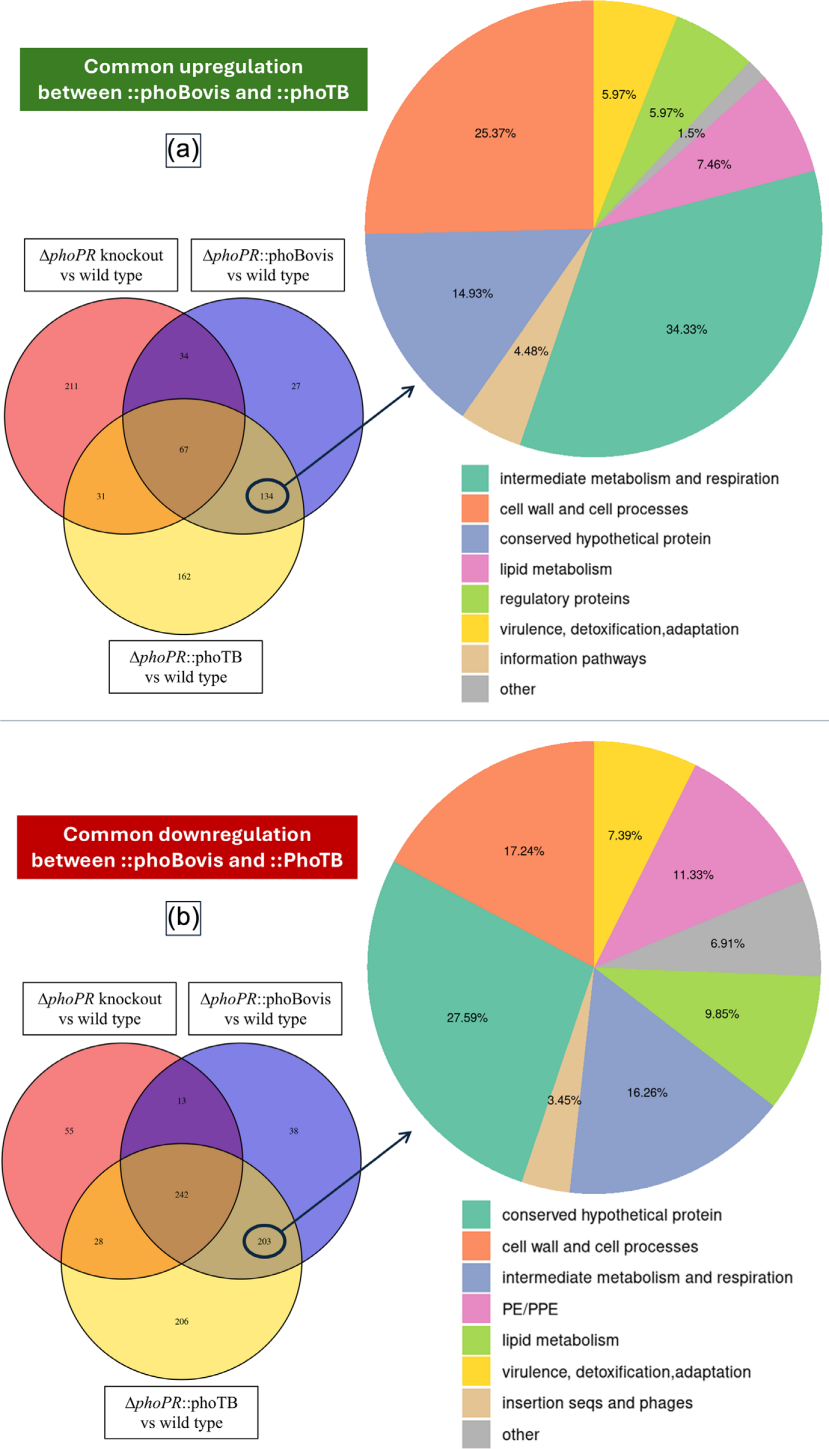
Commonly regulated genes when comparing *M. bovis* Δ*phoPR*::*phoBov* (*phoBovis*) and *M. bovis* Δ*phoPR*::*phoTB* (*phoTB*). Panel (a) shows genes that are commonly upregulated in both strains, and panel (b) shows genes commonly downregulated. On each panel, the Venn diagram shows the intersection of common genes between different comparisons (wild-type vs. *phoPR* knockout, wild-type vs. ::*phoBovis* and wild-type vs. ::*phoTB*). The pie chart zooms in on the comparison between the two complemented mutants and shows the gene categories of the commonly regulated genes.

 We compared the differential expression of the *M. bovis phoPR* knockout and the two complemented mutants expressing the PhoPR orthologues of *M. tuberculosis* or *M. bovis* carrying the G71I substitution, each of them relative to the *M. bovis* wild-type. The first thing we observed was that there is a common transcriptional profile between the *M. bovis* Δ*phoPR*::*phoBovis* and * M. bovis* Δ*phoPR*::*phoTB* complemented mutants (Fig. 1). Also, the expression of *phoP* and key *phoP*-regulated genes [*pks3* (*Mb1213*), *mmpl10* (*Mb1215*), *fadD21* (*Mb1217c*), *Mb1553c*, *pks5* (*Mb1554c*) and *cadI* (*Mb2674*)] was restored on the complement mutants expressing the *M. bovis phoPR* system compared to *M. bovis phoPR* knockout (Supplementary Data file–sheet 6).

Using a threshold of |log_2_fc|>1 and *Padj*<0.05, we observed numerous commonly regulated genes between the complemented mutants expressing either the PhoPR system of *M. bovis* or that of *M. tuberculosis*. In total, 134 genes were commonly upregulated by the two PhoPR systems (Fig. 1a), representing 51.2% of the total upregulated genes in *M. bovis ΔphoPR*::*phoBov* compared to the wild-type (blue panel on the Venn diagram in Fig. 1a) and 34% of the total upregulated genes in *M. bovis ΔphoPR*::*phoTB* compared to the wild-type (yellow panel on the Venn diagram in Fig. 1a). On the other hand, 203 genes were commonly downregulated by the two systems (Fig. 1b), which represent 40.9% of the downregulated genes in *M. bovis ΔphoPR*::*phoBov* (blue panel on the Venn diagram in Fig. 1b) and 29.9% of the downregulated genes in *M. bovis ΔphoPR*::*phoTB* (yellow panel on the Venn diagram in Fig. 1b).

The gene categories of these commonly regulated genes were assessed to look for similar processes that were controlled by both *phoPR* alleles, based on the gene classification of Cole *et al*. [21]. Without taking into consideration conserved hypothetical proteins, for which functional annotations are not available, the processes commonly regulated by the two PhoPR orthologues involved cell wall processes and intermediate metabolism related to respiration and lipid metabolism, systems that are known to be regulated by the PhoPR system of *M. tuberculosis*.

### Differential expression of rubredoxin genes and lipid metabolism pathways

**Fig. 2.**
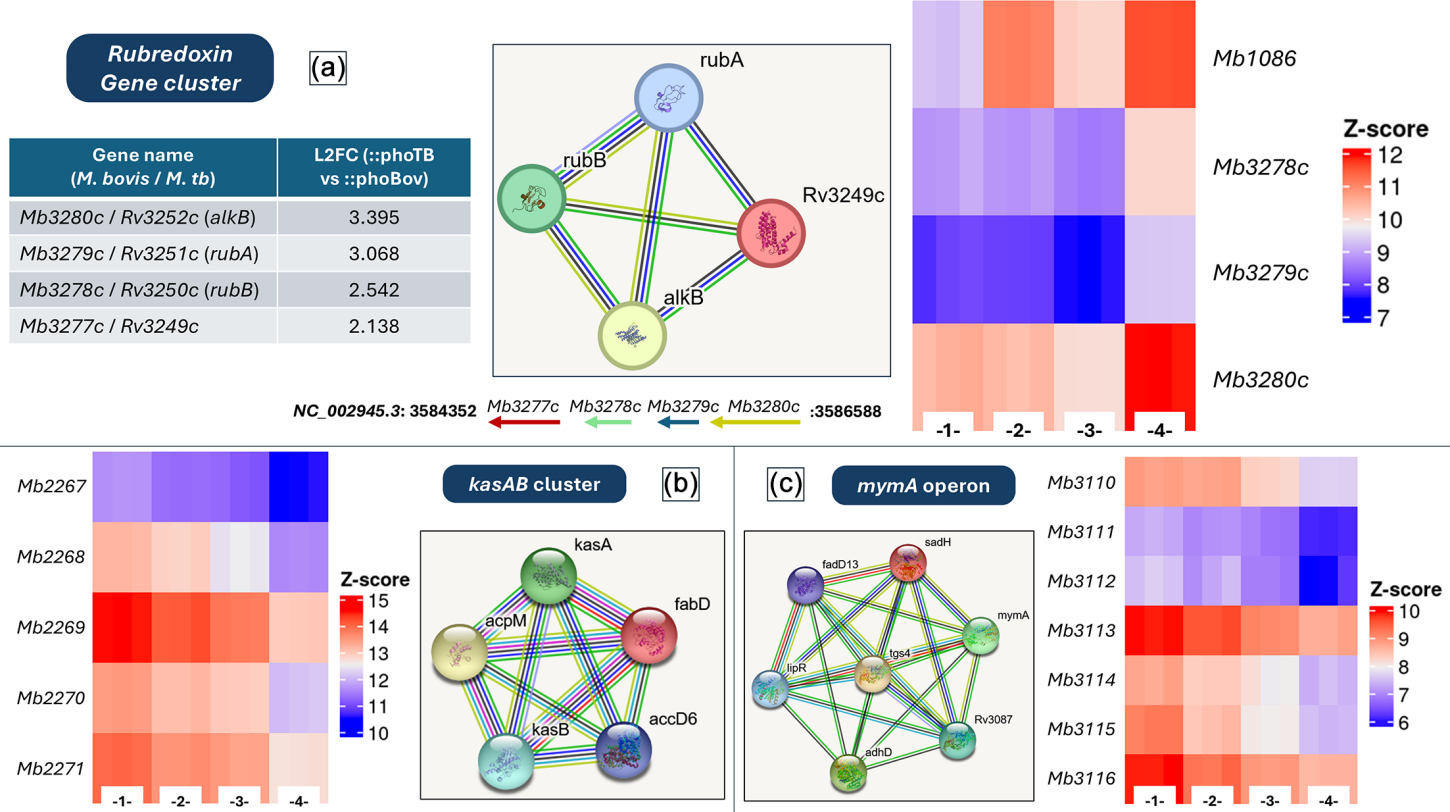
Gene clusters most differentially expressed between *M. bovis ΔphoPR*::*phoBov* and *M. bovis ΔphoPR*::*phoTB*. Panel (a) shows rubredoxin genes, their genetic location and the log_2_ fold change expression on *M. bovis ΔphoPR*::*phoTB* compared to *M. bovis ΔphoPR*::*phoBovis*. Panel (b) shows the *kasAB* cluster, and panel (c) shows the *mymA* operon. The heatmaps show the expression levels of the genes in each cluster between *M. bovis* wild-type (1), *M. bovis* ΔphoPR knockout (2), *M. bovis ΔphoPR*::*phoBov* (3) and *M. bovis ΔphoPR*::*phoTB* (4). The networks represent known gene interactions from the STRING database. The Z-scores represent normalized gene expression levels calculated as described in the methods section.

After observing the common transcription profile between the two complemented mutants, we assessed the key regulatory differences between the two PhoPR orthologues. Three gene clusters stood out as the most differentially expressed loci with statistical significance (*Padj*<0.05), being upregulated on the complement mutants expressing the PhoPR system of *M. tuberculosis*: the rubredoxin gene cluster (Fig. 2a), the *kasAB* cluster (Fig. 2b) and the *mymA* operon (Fig. 2c).

The most differentially expressed genes (DEGs) between the two complements were the three proximate genes *rubB* (*Mb3278c*), *rubA* (*Mb3279c*) and *alkB* (*Mb3280c*). The genes *rubA* and *rubB* encode rubredoxins, which are electron transfer metalloproteins induced by acid stress and iron starvation [26]. These two rubredoxin genes were the most upregulated genes in *M. bovis ΔphoPR*::*phoTB* compared to *M. bovis ΔphoPR*::*phoBovis*, exhibiting an overexpression with a |log_2_fc|>2.5. In the same manner, *alkB*, which encodes a transmembrane protein belonging to the family of alkane/alkyl oxygenases, was also upregulated in * M. bovis ΔphoPR*::*phoTB* with a |log_2_fc|>3. These latter proteins are enzymes that remove alkyl groups from nucleotide bases and are normally involved in DNA repair [27]. These genes are located at the same chromosomal locus as the putative transcriptional regulator *Mb3277c*; we refer to this locus as the rubredoxin gene cluster, which showed the highest differential expression between the two complements, being upregulated by the *M. bovis* mutant expressing the PhoPR system of *M. tuberculosis* (Supplementary Data file–sheet 4).

The two gene clusters involved in lipid biosynthesis were downregulated in *M. bovis* by the *M. tuberculosis* PhoPR orthologue. The *mymA* operon (*Mb3110*–*Mb3116*) consists of the monooxygenase *mymA* (*Mb3110*) gene, followed by several enzymes involved in the condensation of fatty acids into more complex molecules such as keto-acids. These products serve as the building blocks for the biosynthesis of larger molecules, named meromycolic acids, which serve as mycolic acid precursors for cell wall biosynthesis. As with other cell wall lipids, these mycolic acids have both structural and virulence functions [28]. The *kasAB* gene cluster (*Mb2268*–*Mb2271*) includes genes encoding the ketoacyl synthases KasA (*Mb2269*) and KasB (*Mb2270*) in addition to the acyl carrier protein AcpM (*Mb2268*), which are involved in the biosynthesis of meromycolic acids that make up the mycolic acids present in the cell wall of *M. bovis* [29]. Upstream of *kasA* is the gene encoding the malonyl-CoA transacylase FabD (*Mb2267*) and downstream is the carboxyltransferase AccD6 (*Mb2271*) [30]. AccD6 is a key enzyme for mycolic acid biosynthesis that has been shown to be required for pathogenic mycobacteria but non-essential in non-pathogenic mycobacterial species [31].

Taken together, these data suggest that the PhoPR system of *M. tuberculosis* controls the synthesis of mycolic acids in a different manner to the *M. bovis phoPR* orthologue, as these two gene clusters show considerable downregulation in the *M. bovis* complemented mutant expressing the *M. tuberculosis phoPR* orthologue as compared to the *M. bovis* mutant complemented with the *M. bovis phoPR*.

### The PhoPR system of *M. tuberculosis* differentially regulates genes involved in the methylcitrate cycle

**Fig. 3.**
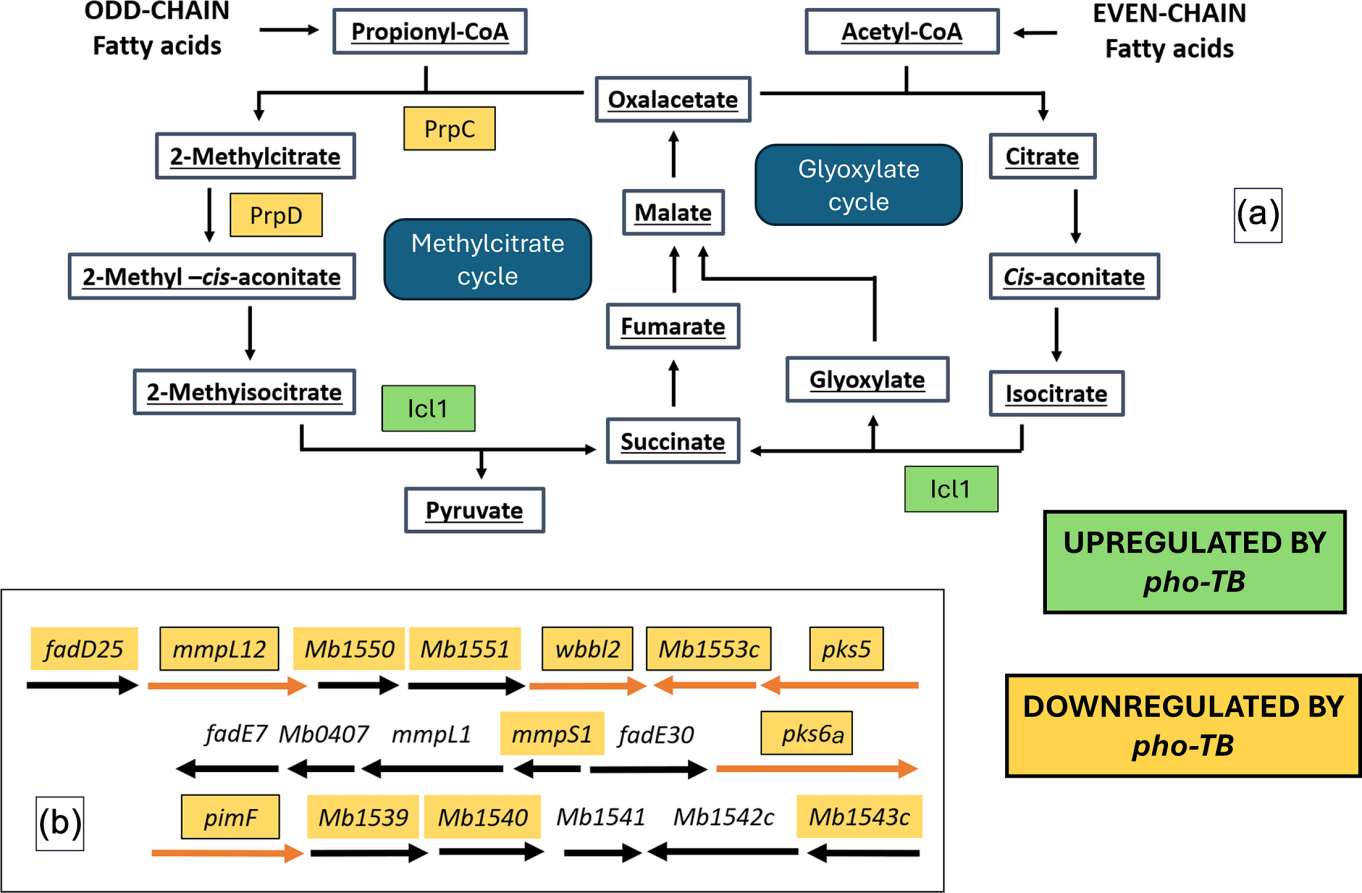
Genes involved in lipid biosynthesis that are DE in *M. bovis* Δ*phoPR*::*phoBov* and *M. bovis* Δ*phoPR*::*phoTB*. Panel (a) shows the metabolic interplay of the methylcitrate cycle and the glyoxylate cycle. Panel (b) shows *pks/mmpL* loci that are DEGs. Genes expressed at lower levels by the *M. tuberculosis phoPR* orthologue are shown in orange, and those expressed at higher levels are shown in green. Orange arrows in panel (b) indicate a |log_2_fc|>1.

Another difference between the two PhoPR orthologues that caught our attention was observed in the expression of genes belonging to the methylcitrate cycle, which is a metabolic pathway involved in the processing of propionyl-CoA that results from the use of fatty acids as a carbon source. Fig. 3 shows the interplay between the methylcitrate cycle and the glyoxylate cycle, highlighting the DEGs in the *M. bovis phoPR* mutant complemented with the *M. tuberculosis phoPR* orthologue compared to the *M. bovis phoPR* mutant expressing the *M. bovis phoPR* orthologue (Fig. 3a). Additionally, the *pks*/*mmpL* loci that were differentially controlled by the two PhoPR orthologues are also shown (Fig. 3b).

Three genes encoding for enzymes involved in the methylcitrate and glyoxylate pathways were differentially expressed in our RNA-seq data when comparing *M. bovis* Δ*phoPR*::*phoBovis* and *M. bovis* Δ*phoPR*::*phoTB*. Two of these DEGs, *prpC* (*Mb1162*) and *prpD* (*Mb1161*), are specific to the methylcitrate cycle and encode a methylcitrate synthase and a methylcitrate dehydratase, respectively. These genes were expressed at lower levels by the *M. tuberculosis phoPR* allele. On the other hand, the bifunctional enzyme encoded by *icl1* (*Mb0476*), which acts as a 2-methylisocitrate lyase in the methylcitrate cycle and as an isocitrate lyase (ICL) in the glyoxylate cycle, was expressed at a higher level in *M. bovis* Δ*phoPR* complemented by the *M. tuberculosis phoPR* system compared to the *M. bovis phoPR*.

## Discussion 

Our study showed clear differences between the transcriptional profile of *M. bovis* expressing either the PhoPR system of * M. bovis* or *M. tuberculosis*. The biggest difference was found for the rubredoxin locus, where the genes *rubA* and *rubB* are in an operon with the alkane oxygenase gene (*alkB*) and a putative transcriptional regulator *Mb3277c* upstream of *rubA*. All these genes are upregulated in the *M. bovis* complement expressing the *M. tuberculosis* PhoPR orthologue as compared with the * M. bovis* orthologue. Rubredoxins are iron-sulphur-containing proteins that play a role in responding to oxidative stress; they have been suggested to play an important role in *M. tuberculosis* adaptation to the intracellular environment by maintaining redox homeostasis [26]. Our data suggest a differential control of the expression of this rubredoxin gene cluster by the PhoPR system of *M. tuberculosis* as compared to that of *M. bovis*. In addition to these genes, one other gene showed a |log_2_fc|>2.5 on the complemented mutant expressing the PhoPR allele of *M. tuberculosis*, which was *Mb1086*. This is induced by acid shock in *M. tuberculosis* [32], and it encodes the only β-propeller protein in *M. bovis*. The β-propeller proteins have a range of functions, and the homologous gene to *Mb1086* in *M. tuberculosis*, *Rv1057*, has been suggested to play a role in the secretion of the major virulence factor ESAT-6 [33]. Since ESAT-6 is dependent on the presence of PhoPR in *M. tuberculosis* [10], and *M. bovis* has been observed to differently regulate the expression of ESX-1 proteins compared to *M. tuberculosis* [34], these differences could also be linked to distinct host-pathogen interaction strategies.

The PhoPR system controls various lipid metabolic pathways, including the expression of genes involved in the biosynthesis of the mycobacterial cell wall lipids, sulpholipid-1, polyacyltrehaloses and phosphatidylinositol mannoside [35]. A previous comparative study showed differences in host-pathogen interactions between *M. bovis* and *M. tuberculosis* and discussed the implication of cell wall lipid differences in the virulence of these two pathogens [34]. The gene *pks5* (*Mb1554c*) was also one of the most downregulated on *M. bovis* Δ*phoPR*::*phoTB* compared with the complement mutant expressing the PhoPR of * M. bovis*. Moreover, *lipR* (*Rv3084*/*Mb3111*) and *sadH* (*Rv3085*/*Mb3112*), which are part of the *mymA* operon [28], have previously been identified as being expressed at lower levels in the attenuated *M. tuberculosis* H37Ra strain compared with the virulent *M. tuberculosis* H37Rv [36]. Since these genes are also expressed at lower levels in our complemented mutants expressing the *M. tuberculosis phoPR* orthologue, this could also point to the different host tropism of *M. bovis* and *M. tuberculosis*. We also observed differences in the expression of genes involved in fatty acid metabolism. Evidence suggests that fatty acids are the preferred carbon source for intracellular growth of *M. tuberculosis*. The assimilation of fatty acids renders propionate as a metabolic by-product that can be further degraded through different metabolic processes [28,29, 37]. In addition to incorporating the propionyl-CoA into the mycobacterial cell wall in the form of methyl-branched lipids, *M. tuberculosis* is known to rely on two different strategies for the detoxification of propionyl-CoA. One of them is the methylcitrate cycle, and the other is the vitamin B12-dependent methylmalonyl pathway [38]. The ICLs of *M. tuberculosis* have been shown to be essential for growth on acetate and propionate because of their dual activity, and the lack of these enzymes converts the methylcitrate cycle into a ‘dead end’ pathway. The activation of the B12-dependent methylmalonyl pathway is an alternative metabolic strategy that has been seen to partially compensate for the lack of ICLs, but the growth of ICL-deficient *M. tuberculosis* was still restricted [39]. The DE genes *icl1*, *prpC* and *prpD* seen in our *M. bovis* △*phoPR* complemented with the different *phoPR* orthologues were an interesting observation because these three enzymes had previously been identified as involved in the adaptation of *M. tuberculosis* to acid environments on different carbon sources. Transcriptional profiling of *M. tuberculosis* revealed that *icl1* was induced at acidic pH, and the two methylcitrate specific enzymes PrpC and PrpD were repressed in the presence of pyruvate but induced during growth on glycerol. Other enzymes related to anaplerotic carbon metabolism were also induced during acidic growth [6,37]. These findings all point to the potential for the point mutant differences between the *M. bovis* and *M. tuberculosis* PhoPR systems as being ways to fine-tune the downstream expression of the regulon’s genes, which may, in turn, serve to optimize metabolism and virulence of the different bacteria for their respective hosts.

The degree of differential expression between the *M. bovis* and the *M. tuberculosis phoPR* alleles may be in some ways surprising, given that the PhoP transcriptional regulator has an identical sequence between the two species. While changes in PhoR may alter sensing of signals through the system, once ‘triggered’, the PhoP regulator would be expected to bind/control the same sets of genes. PhoP can directly bind to about 35 loci in the *M. tuberculosis* genome [40]. The differential expression seen with PhoPR orthologues in our work may therefore suggest a more nuanced role of PhoR variants in control of PhoP and the regulon. As Chiner-Oms *et al*. highlighted, mutations of the PhoR kinase could act to fine-tune virulence and host-pathogen interaction [41]. Our findings align with this idea of kinase variants potentially linking to host specificity.

**Fig. 4.**
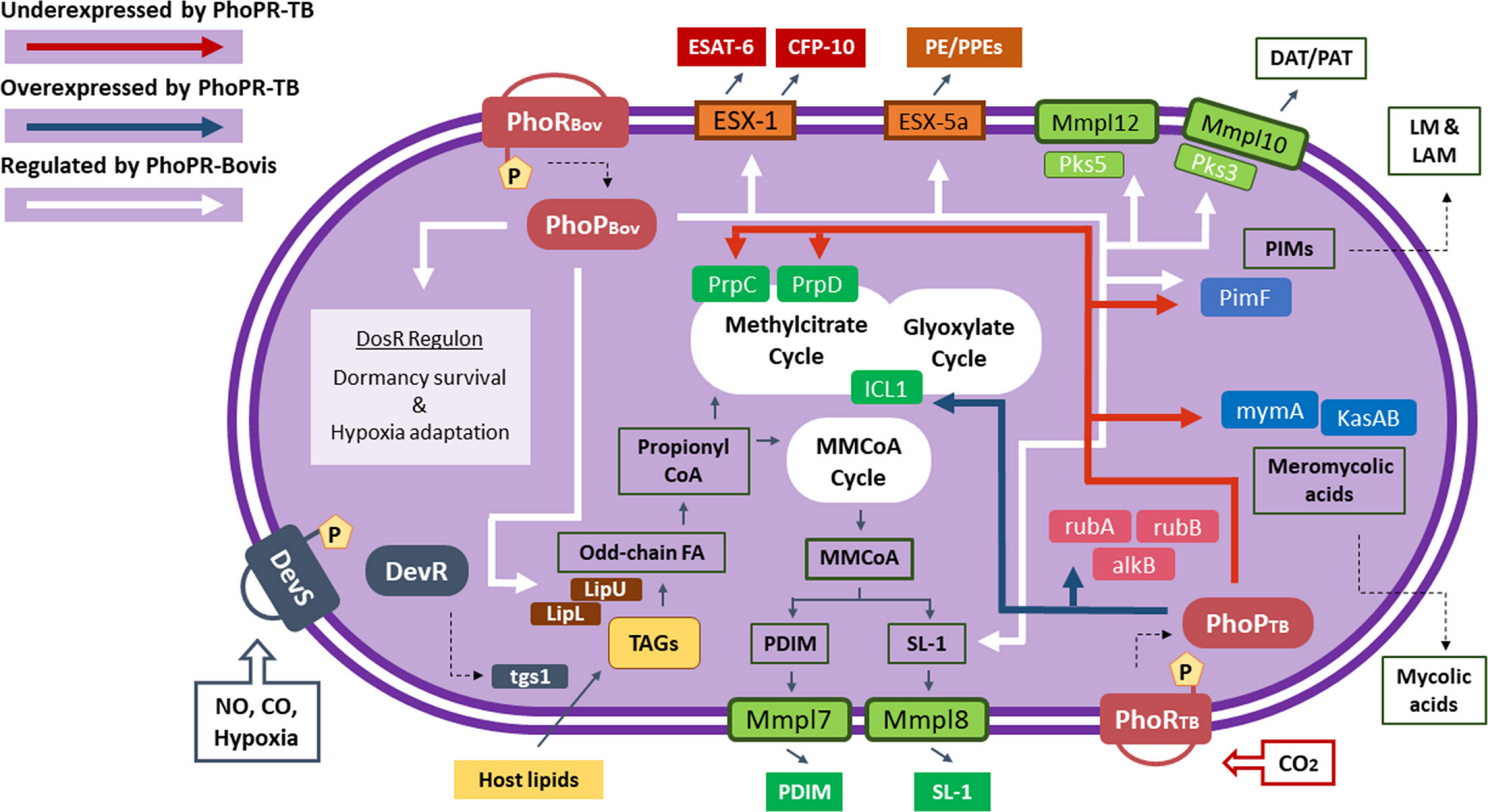
Schematic representation of the mechanisms that are controlled by the PhoPR system of *M. bovis* or *M. tuberculosis*. White arrows represent processes controlled by the *M. bovis* PhoPR system, as found with the DEG analysis of the *M. bovis* ΔphoPR compared to *M. bovis* wild-type. Blue arrows correspond to processes upregulated by the presence of the PhoPR system of *M. tuberculosis* found by comparing the complemented mutants expressing either of the two PhoPR orthologues, and red arrows represent the processes downregulated by the *M. tuberculosis* PhoPR system.

The differential regulation of the rubredoxin gene cluster (*Mb3277c*–*Mb3280c*) in *M. bovis* expressing the *M. tuberculosis phoPR* orthologue is, to our knowledge, the first identification of PhoPR controlling these genes. In addition to the rubredoxin gene cluster, complementation with the *M. tuberculosis* PhoPR system led to differential expression of two gene clusters involved in the production of mycolic acids, the *mymA* operon (*Mb3110*–*Mb3116*) and the *kasAB* gene cluster (*Mb2267*–*Mb2271*). Rubredoxins have been associated with the *M. tuberculosis* stress response [26], and mycolic acids are known to have both structural and pathogenic roles [28,29]; the difference observed in the expression of these genes by the *M. bovis* or *M. tuberculosis* PhoPR system may also indicate a difference between the pathogens in stress responses and intracellular adaptation. Fig. 4 summarizes the findings of this study together with the transcriptional profile of the *M. bovis* Δ*phoPR* knockout, providing an overview of the complex mechanisms underlying the PhoPR system of *M. bovis* compared to the PhoPR from *M. tuberculosis*.

## Supplementary material

10.1099/acmi.0.001087.v3 Uncited Supplementary Material 1.

## Abbreviations

DEGs: differentially expressed genes
ICL: isocitrate lyase
MTBC: *Mycobacterium tuberculosis* complex
*Padj*: *P*-adjusted value
RNA-seq: RNA sequencing
TB: tuberculosis
